# Comparative transcriptome analysis in peaberry and regular bean coffee to identify bean quality associated genes

**DOI:** 10.1186/s12863-022-01098-y

**Published:** 2023-02-27

**Authors:** Xingfei Fu, Guiping Li, Faguang Hu, Jiaxiong Huang, Yuqiang Lou, Yaqi Li, Yanan Li, Hongyan He, YuLan Lv, Jinhuan Cheng

**Affiliations:** Institute of Tropical and Subtropical Cash Crops, Yunnan Academy of Agricultural Sciences, Yunnan Baoshan, 678000 China

**Keywords:** *Coffea arabica*, Transcriptome analysis, Gene expression, Bean quality, Bean components

## Abstract

**Background:**

The peaberry bean in Arabica coffee has exceptional quality compared to the regular coffee bean. Understanding the molecular mechanism of bean quality is imperative to introduce superior coffee quality traits. Despite high economic importance, the regulatory aspects of bean quality are yet largely unknown in peaberry. A transcriptome analysis was performed by using peaberry and regular coffee beans in this study.

**Results:**

The result of phenotypic analysis stated a difference in the physical attributes of both coffee beans. In addition, transcriptome analysis revealed low genetic differences. Only 139 differentially expressed genes were detected in which 54 genes exhibited up-regulation and 85 showed down-regulations in peaberry beans compared to regular beans. The majority of differentially expressed genes had functional annotation with cell wall modification, lipid binding, protein binding, oxidoreductase activity, and transmembrane transportation. Many fold lower expression of *Ca25840-PMEs1, Ca30827-PMEs2, Ca30828-PMEs3, Ca25839-PMEs4, Ca36469-PGs.* and *Ca03656-Csl* genes annotated with cell wall modification might play a critical role to develop different bean shape patterns in Arabica. The ERECTA family genes *Ca15802-ERL1, Ca99619-ERL2, Ca07439-ERL3, Ca97226-ERL4, Ca89747-ERL5, Ca07056-ERL6, Ca01141-ERL7*, and *Ca32419-ERL8* along lipid metabolic pathway genes *Ca06708-ACOX1, Ca29177-ACOX2, Ca01563-ACOX3, Ca34321-CPFA1*, and *Ca36201-CPFA2* are predicted to regulate different shaped bean development. In addition, flavonoid biosynthesis correlated genes *Ca03809-F3H*, *Ca95013-CYP75A1*, and *Ca42029-CYP75A2* probably help to generate rarely formed peaberry beans.

**Conclusion:**

Our results provide molecular insights into the formation of peaberry. The data resources will be important to identify candidate genes correlated with the different bean shape patterns in Arabica.

**Supplementary Information:**

The online version contains supplementary material available at 10.1186/s12863-022-01098-y.

## Background

Coffee is one of the most popular beverages nowadays. Millions of people in the world consumed coffee to boost their concentration, productivity, and physical performance [1]. It becomes prime source of income in tropical regions of different countries, produced almost seven million tons every year worldwide, and is ranked among the top five most agricultural export commodities of devolving countries [2]. The Brazil, Vietnam, and Colombia produced more than 50% of global coffee. The countries such as China, Ethiopia, Honduras, Indonesia, India, Malaysia, Nicaragua, and Peru are other major coffee growing countries in the world. Moreover, rigorous consumption of coffee beverages and their commercialization ultimately caused wide development of the coffee industry in many non-tropical countries in recent years [3–5]. In China, the successful cultivation of coffee was first reported in Taiwan province followed by Yunnan province and the tropical area of Hainan province [6]. The genus of *Coffea* has 124 species with the addition of 20 closely related species from the genus *Psilanthus* [7]. However, *Coffea arabica* (Arabica) and *C. canephora* (Robusta) have more economic importance which generates 70% and 30% of world coffee production, respectively [8]. The Arabica is allotetraploid species with 2*n* = 4× = 44, well adapted to highlands, and proved to have the best quality coffee beans than other species. In contrast, Robusta is diploid species with 2*n* = 2x = 22, better adaptation to warm or humid climatic conditions of lowlands, and regarded low quality coffee than Arabica due to higher caffeine concentration in beans [9–11]. Climate change and insect pest resilient genotypes are critical to mitigating the recent decline of coffee productivity worldwide [12].

With the increased knowledge of quality characteristics among consumers, the demand for high quality coffee beans has been increasing. The regular consumption of quality coffee usually not only improves physical performance but also reduces the risk of various disorders [13]. The Arabica coffee has aluminous dicots bean with various stored compounds in the mature endosperm [14]. The cell wall polysaccharides, sucrose, lipids, proteins, and chlorogenic acids are major storage compounds present in mature green coffee beans [15–18]. The precursor of these compounds determines the coffee final aroma, flavor, and taste [19]. The biochemical composition of storage compounds alters with environmental variables and genotypes [20]. A better understanding of the molecular mechanism of bean quality has critical importance to breed high quality coffee genotypes. The recent development in transcriptomic, proteomic, and metabolomics analytical techniques has identified the candidate genes related to bean storage components in different crops [21–23]. The high throughput research on coffee crops has gained attention with the recent free availability of the Robusta reference genome [24] and the draft genome of Arabica [25]. Different studies have already been performed to investigate the genetic control of various stress resistance [26, 27] and the accumulation of major bean components in various species of coffee [28, 29]. However, a significant research gap still exists in Arabica. The genetic mechanism of bean quality traits is limited in Arabica. Large-scale high-throughput transcript data resources can help to hybridize high quality bean genotypes in Arabica.

The coffee plant produced fruit cherries and beans are the seeds inside ripened fruit. Usually coffee fruit cherry has two embryos, their fertilization generate two independent hemispherical shape beans. However, sometimes only one embryo is further developed to yield round shape thicker bean which is commonly known as a peaberry [30]. The probability of peaberries occurrence is extremely low under normal conditions. Almost, 7% of mature green coffee crop is comprised of peaberries. The peaberries are rare in nature and can be formed at any pace in coffee planting areas [31]. The bean physical attributes is prime trait that not only disturbs market price but also significantly affects coffee roasting time [32]. To ensure high coffee quality, the customers commonly separate peaberries from regular beans due to their higher market price and cup quality. Because of economic importance of peaberries, this study was designed to fulfill the research gap existing for peaberry bean quality traits. The beans physical attributes such as single bean size, length, and width were measured by using peaberry and regular coffee beans. Furthermore, a comparative transcriptome analysis was performed to reveal gene expression differences between both coffee beans. The results of this study further provide molecular insights into bean quality traits of peaberry coffee.

## Results

### Phenotypic shape differences among peaberry and regular coffee beans

**Fig. 1:**
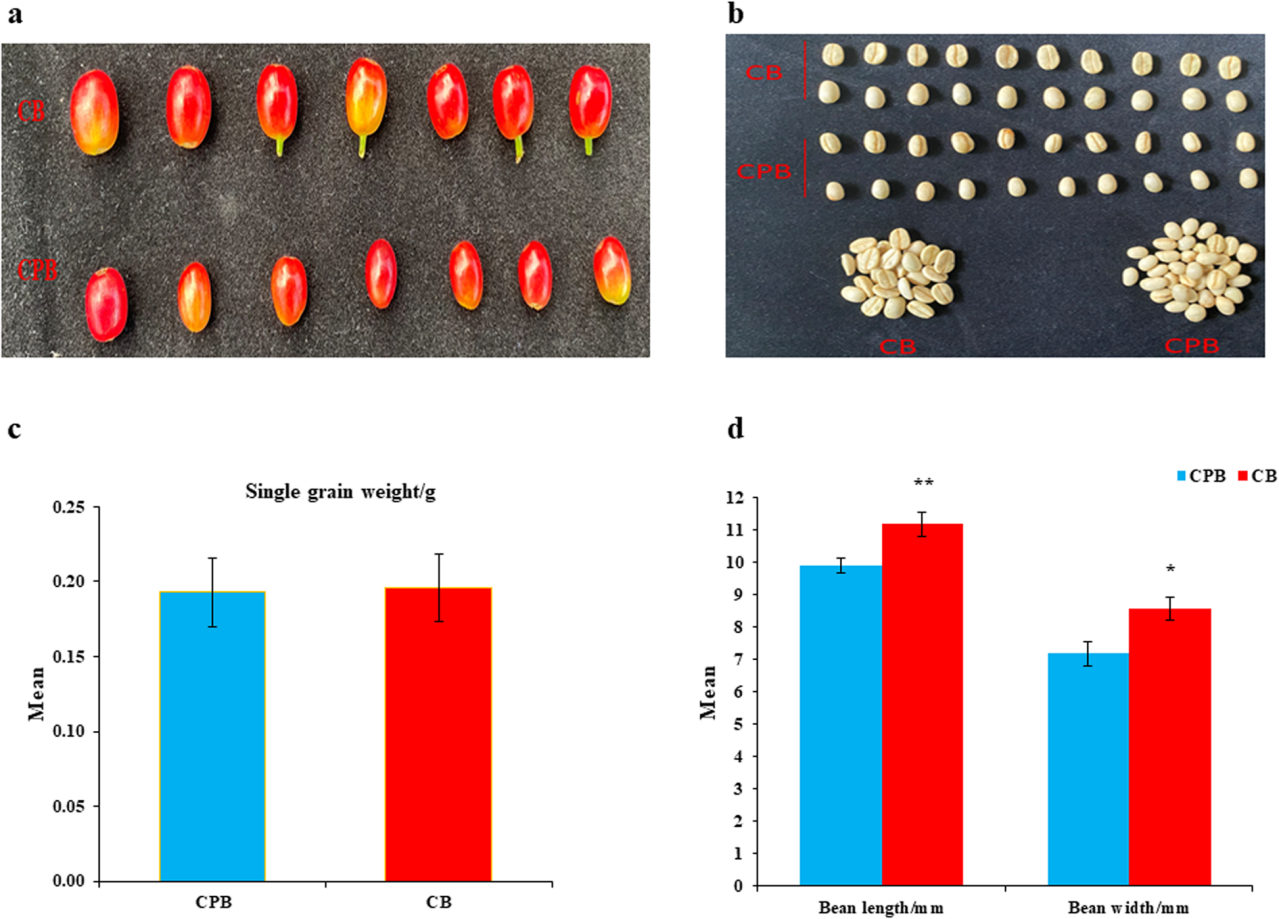
The phenotypic difference among peaberry and regular coffee beans **a** Mature of peaberry coffee beans (CPB) and regular coffee beans (CB), **b** Front and back view of CPB beans, front and back view of CB beans, **c** Mean comparison of single bean weight, **d** Mean comparison of single bean length and width among CPB and CB. ** is used for significant difference at *p < 0.01* and * at *p < 0.05*

The ripened fruit of Arabica generally contains two regular bean seeds. The probability of occurrence of peaberry coffee beans is extremely low. This study determined the phenotypic attributes of peaberry and regular coffee beans. Hereafter, these contrasting coffee beans were named CPB (peaberry coffee bean) and CB (regular coffee bean). Interestingly, mature fruit cherry of CPB has a different shape compared to CB (Fig. 1a). The peeled bean of peaberry is round shaped whereas regular beans had hemispherical shape (Fig. 1b). The average of 20 beans showed that CPB and CB had significant difference in bean length and width. However, the single bean weight had non-significant difference. The mean value of single bean weight was 0.19 g for CPB whereas CB had mean value of 0.20 g in this study (Fig. 1c). The bean length and width for CB had a mean of 11.19 mm and 8.56 mm, respectively. However, the bean length and width of CPB were somehow lower with the observed mean of 9.9 mm and 7.19 mm, respectively (Fig. 1d). These results revealed that peaberries had contrast bean shape as well as physical attributes in comparison to regular coffee beans.

### Overview of transcriptome sequencing in peaberry and regular coffee beans

In this study, high throughput RNA sequencing was achieved in three biological repeats for each coffee bean. Then, comparative transcriptome analysis was performed between CPB and CB to explore the regulatory genes associated with bean quality of peaberry. The transcriptome analysis revealed that total sequenced bases, total reads, and clean reads were relatively higher in CB as compared to CPB. The mean of total sequenced bases was 11,415,771,900 in CPB wherein the total reads, and clean reads mean were 76,105,146, and 76, 047,177, respectively (Table 1). In contrast, the total sequenced bases mean was 13,641,586,400 for CB with total reads and clean reads mean of 90, 943,909 and 90,873,708, respectively. Almost, the 93% of clean reads were mapped to reference the *genome* of *C. arabica*. Of which, nearly 75% of reads were uniquely mapped and only 18% were multiple mapped. The mean of Q30 was above 93% for each sequenced sample. Approximately, 88% of reads were mapped to the exon region in both coffee beans whereas intronic, intergenic, and splicing were almost 6%, 4%, and 1%, respectively (Figure S1). All these results state high quality sequenced data suitable for downstream analysis. The principal component analysis (PCA) revealed that the PC1 and PC2 described 58% of the total variation among all samples (Figure S2a). The statistics of correlation analysis stated undulant correlations among different samples of both coffee beans (Figure S2b).

**Table 1:** Overview of the transcriptome sequencing and quality parameters for peaberry and regular coffee beans

Samples	Total bases	Total reads	Clean reads	Mapped Reads	Multiple mapped (%)	Uniquely mapped (%)	Q30 (%)
CPB1_1	11,829,184,200	78,861,228	78,801,468	93.6	19.7	73.9	93.9
CPB1_2	11,021,794,500	73,478,630	73,428,842	93.9	18.2	75.7	93.5
CPB1_3	11,396,337,000	75,975,580	75,911,220	93.6	18.6	74.9	93.4
CB1_1	14,501,058,900	96,673,726	96,603,218	93.4	17.7	75.7	93.4
CB1_2	13,622,697,000	90,817,980	90,744,098	93.7	17.9	75.8	93.0
CB1_3	12,801,003,300	85,340,022	85,273,808	94.5	18.9	75.7	94.0

### Differentially expressed genes among peaberry and regular coffee beans

**Fig. 2:**
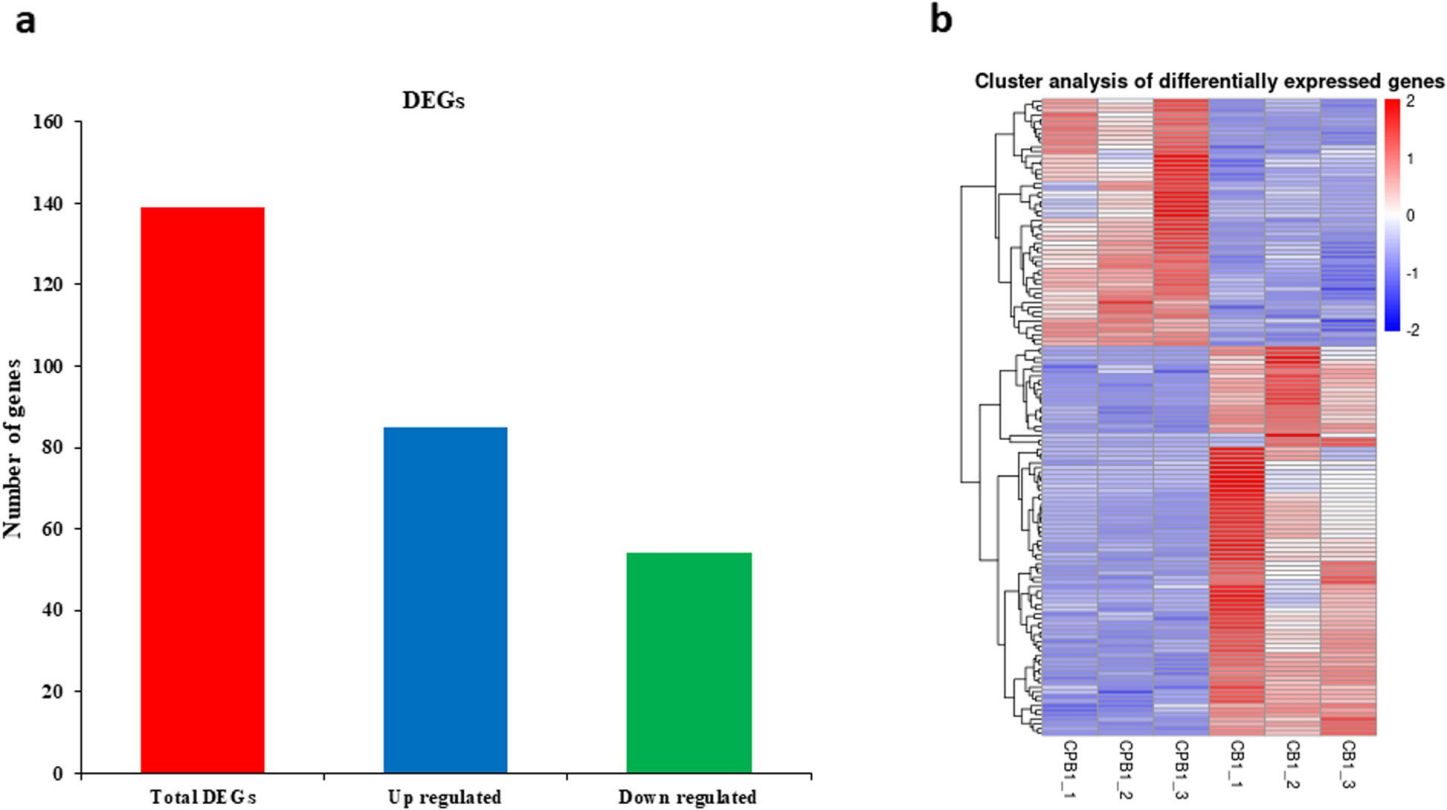
The total DEGs, their regulation, and expression profiles in comparison of peaberry and regular coffee beans **a** Total DEGs distribution **b** Expression profiles of total DEGs in clustered form

The total number of expressed genes describes the overall view of the transcript landscape in the given sample. The expression level was measured with fragments per kilobase per million reads (FPKM) value. Our results found a higher number of total expressed genes for CB than for CPB. For example, the total number of expressed genes was 38,543 for CB (Table S1). However, 37,765 genes were expressed in CPB. The higher ratio of genes had 0.1–3.75 FPKM expression followed by 3.75-15 FPKM in both coffee beans (Table S1). However, the ratio was determined little higher for CB than CPB. The ratio of gene expression with > 15 FPKM value was 14.68% for CB and 14.33% for CPB. The FPKM scores were utilized to analyze the dynamic gene expression differences among CB and CPB. The differentially expressed genes (DEGs) between coffee beans were considered with *p ≤ 0.05* and log2 (fold change) ≥ 1 or log2 (fold change) ≤-1. The total number of DEGs with the distribution of up or down regulation is shown in Fig. 2. Comparative analysis among CB and CPB had shown 139 total DEGs (Fig. 2a) with 85 genes up regulated in CB compared to CPB. In contrast, 54 genes were down regulated in CB compared to CPB. Cluster analysis of the DEGs showed that genes had distinct expression clusters with contrasting expression trends between both coffee beans (Fig. 2b). The lower number of DEGs demonstrated that both coffee beans had the same genetic background but small gene expression profiles led to formation of peaberry coffee beans in Arabica. Functional enrichment analysis showed that most genes were annotated with pectinesterase activity, enzyme inhibitor activity, manganese ion binding, ethylene-activated signaling pathway, and cell wall modification (Figure S3). Therefore, our results presume that these gene dynamic expression changes and interactions influence bean quality traits of peaberry coffee.

### Identification of bean quality traits associated genes in peaberry coffee

**Fig. 3:**
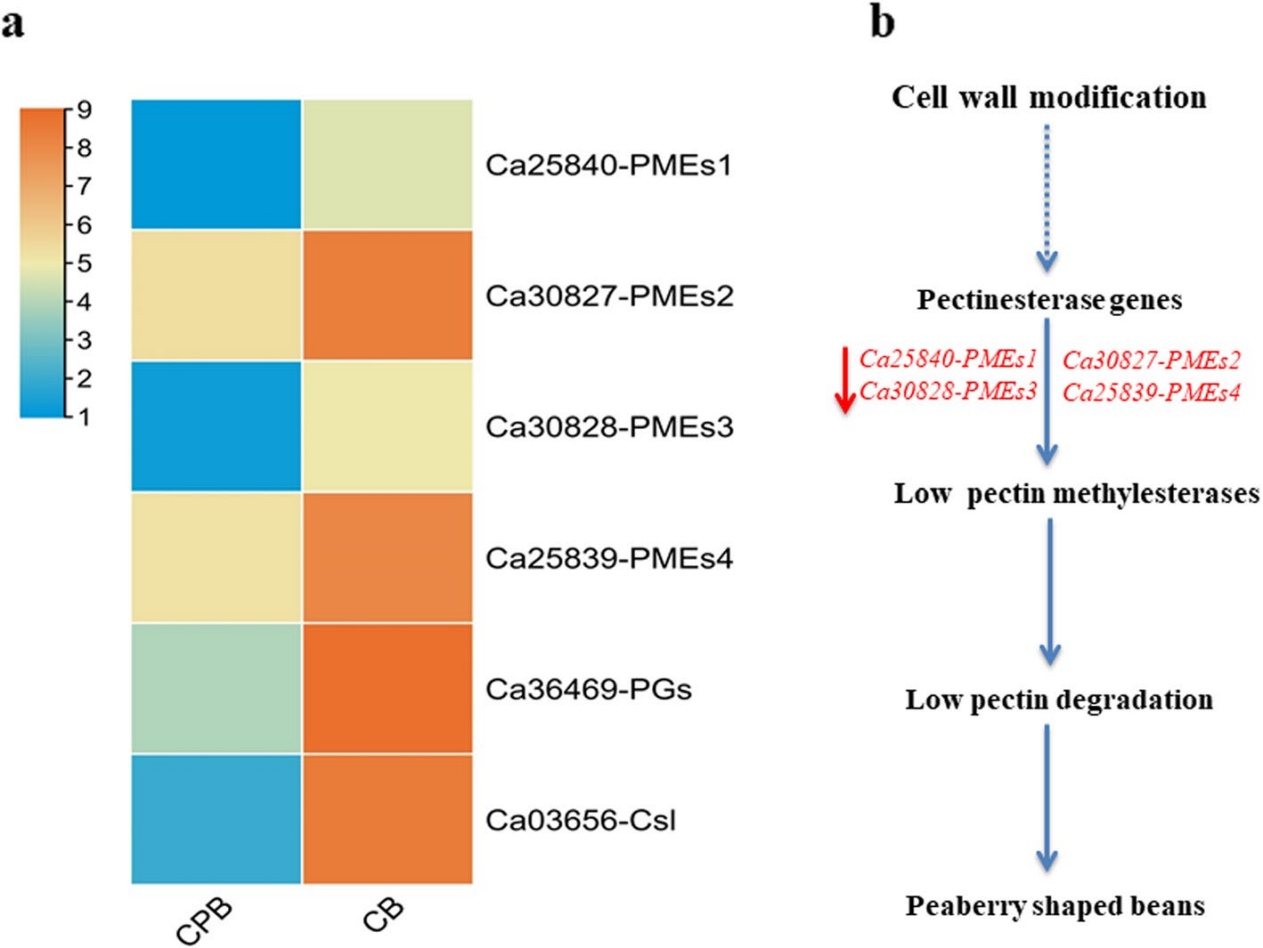
The expression profiles of genes related to cell wall modification and how these regulate bean shape of peaberry **a** Expression profiles among CPB and CB **b** Simplest predicted mechanism of peaberry-shaped beans. The down-regulation of cell wall modification genes in CBP than CB might lead to lower pectin degradation and peaberry-shaped coffee beans. The red arrow represents the down-regulation of expression. PMEs: pectin methylesterases

The matured coffee bean endosperm is comprised of different compositions of cell wall polysaccharides, sucrose, lipids, proteins, and chlorogenic acids [14]. These storage compounds produce coffee color, aroma, and taste through a series of complex chemical reactions on roasting [8]. However, the roasting method in addition to total time had the least effect on the quality traits of coffee beans. Therefore, the exploration of potential genes tightly correlated with quality attributes of matured green beans is essential to improving the quality aspects of coffee. Our targeted analysis identified several important genes associated with bean quality components of peaberry coffee beans. For instance, genes *Ca25840-PMEs1, Ca30827-PMEs2, Ca30828-PMEs3, Ca25839-PMEs4, Ca03656-Csl*, and *Ca36469-PGs* involved in cell wall modification had shown significantly altered expression in the comparison of both coffee beans (Fig. 3a). All these genes were annotated with pectin modifying enzymes such as pectin methylesterases (PMEs) and polygalacturonase (PGs) as well as cellulose synthase-like (Csl). The pectin in addition to cellulose and hemicellulose are major constituents of the cell wall in plants. The degradation of pectin with pectinesterases or polygalacturonase contributes to cell wall plasticity, morphogenesis, intercellular communication, and pollen separation in plants [33–35]. Many fold lower expressions of *Ca30827-PMEs2, Ca25839-PMEs4*, and *Ca36469-PGs* in CPB anticipated their essential role in the modification of cell wall architecture. This modification of cell wall components might play a critical role to develop different bean shape patterns in peaberry coffee (Fig. 3b). However, functional analysis is needed to quantify how these genes interact to induce the formation of peaberry and regular coffee beans.

**Fig. 4:**
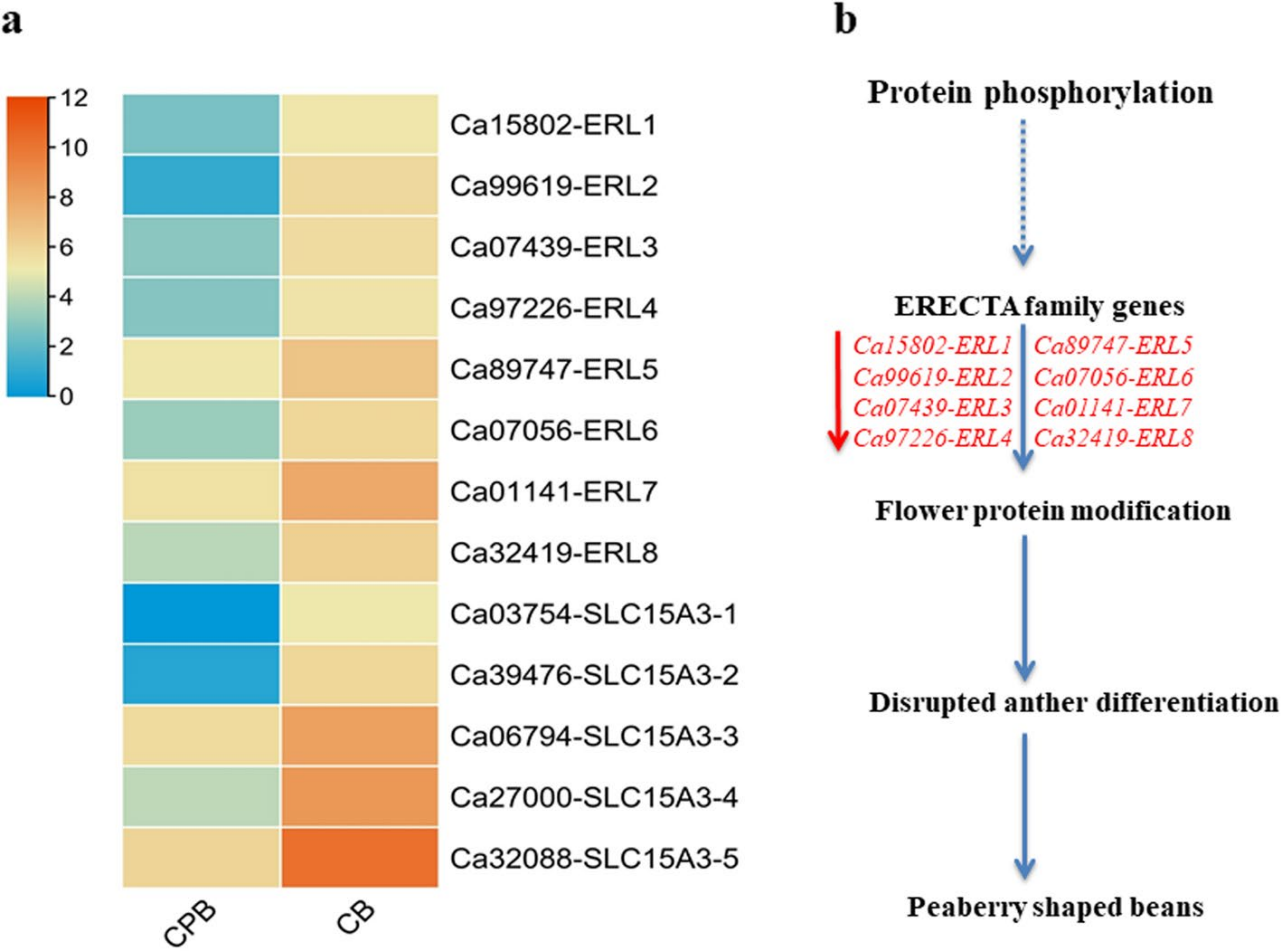
The expression profiles of protein regulatory genes and how these regulate the development of peaberry **a** Expression profiles among CPB and CB **b** Simplest predicted mechanism of peaberry-shaped beans. The down-regulation of ERECTA family genes in CBP than CB leads to disruption in anther differentiation that possibly generates peaberry-shaped coffee beans. The red arrow represents the down-regulation of expression. ERL: ERECTA receptor-like serine/threonine-protein kinase

In addition, our analysis determined that eight DEGs involved in the biological function of protein phosphorylation and belong to LRR receptor-like serine/threonine-protein kinase ERECTA family exhibited different expression profiles in both coffee beans. These genes included *Ca15802-ERL1, Ca99619-ERL2, Ca07439-ERL3, Ca97226-ERL4, Ca89747-ERL5, Ca07056-ERL6, Ca01141-ERL7*, and *Ca32419-ERL8* (Fig. 4a). The *ERL* encoding transcripts have a diverse functional role in plant growth. Their defects produced irregular flower growth, petal polar expansion, carpel elongation, and anther and ovule differentiation in Arabidopsis [36]. In consistent with earlier research, signifcantly lower expression of ERLs related genes may confer disruption in normal flower growth in Arabica which ultimately led to the synthesis of peaberry coffee beans (Fig. 4b). Moreover, protein phosphorylation is a post-translational protein modification. It subsequently facilitates the biosynthesis and degradation of storage protein in plants [37]. Previously, it has been reported that stored proteins convert cell wall polysaccharides and sugars into aroma quality compounds in coffee [19]. In peaberry coffee beans, several solute carrier family 15 encoding genes that include *Ca03754*-*SLC15A3-1, Ca39476*-*SLC15A3-2, Ca06794-SLC15A3-3, Ca27000-SLC15A3-4*, and *Ca32088-SLC15A3-5* and involved in peptide/histidine transportation had significant expression differences among both coffee beans (Fig. 4a). These functional genes are probably interlinked with the transport of reserve protein during flower development. The dynamic changes in genes performed protein functional activities maybe contribute to the development of peaberry coffee beans.

**Fig. 5:**
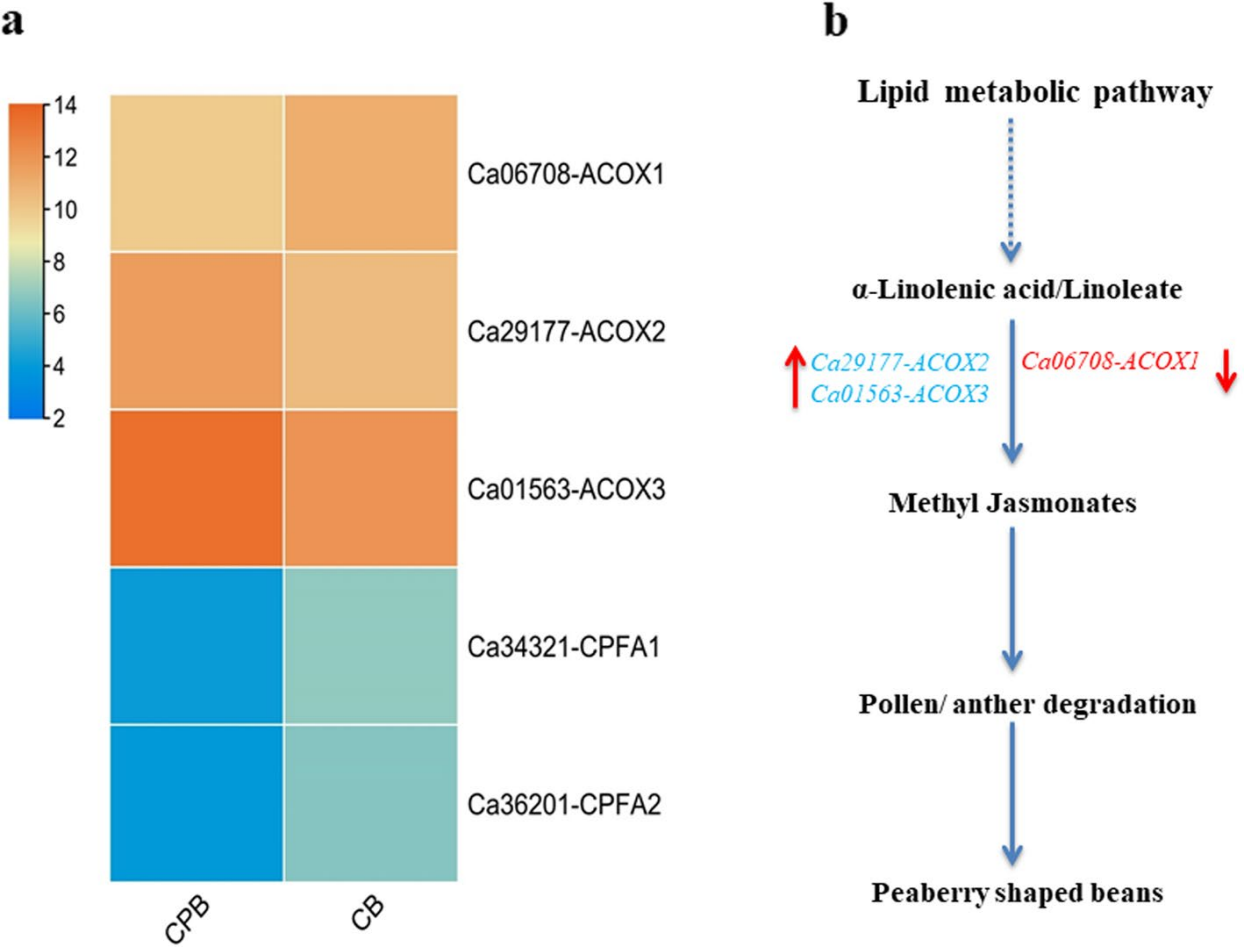
The expression profiles of lipid/fatty acid metabolic genes and how these influence the bean shape of peaberry **a** Expression profiles among CPB and CB **b** Simplest predicted mechanism of peaberry-shaped beans. The altered expression of lipid metabolic genes in CBP than CB might cause pollen degradation that results in peaberry-shaped coffee beans. The red down arrow and red up arrow represent the down-regulation and the up-regulation of expression. ACOX: acyl-CoA oxidase 3

The lipids and fatty acids are essential storage components that also contribute to the sensory quality of coffee beans [38]. Like many other genes, our results found that many lipid metabolisms annotated genes that include acyl-CoA oxidase 3 (*Ca06708-ACOX1, Ca29177-ACOX2*, and *Ca01563-ACOX3)* along with cyclopropane-fatty-acyl-phospholipid synthase (*Ca34321-CPFA1* and *Ca36201)* had shown significant differences among CPB and CB (Fig. 5a, b). In particular, expression of *Ca06708-ACOX1* gene had shown lower expression pattern compared to other two *ACOX* related genes. This predicts their key disrupted role in oxidation of acyl-CoA and most likely associate with the formation of peaberry beans. Previous molecular experimentation has shown that regulatory genes associated with lipids, fatty acids, and their derivatives often play a vital role in the reproductive development of anther and pollen in plants [39]. Altered expression of these genes suggested their essential role in the accumulation of different profiles of lipid and fatty acid in peaberry coffee beans. This might influence the balance of lipids metabolism which results in the formation of peaberry bean in Arabica. Our target analysis further revealed that flavonoids biosynthesis encoding genes such as flavanone-3-hydroxylase (*Ca03809-F3H*) and cytochrome P450 family 75 subfamily A (*Ca95013-CYP75A1* and *Ca42029-CYP75A2*) exhibited significantly lower expression in CPB than CB.

**Fig. 6:**
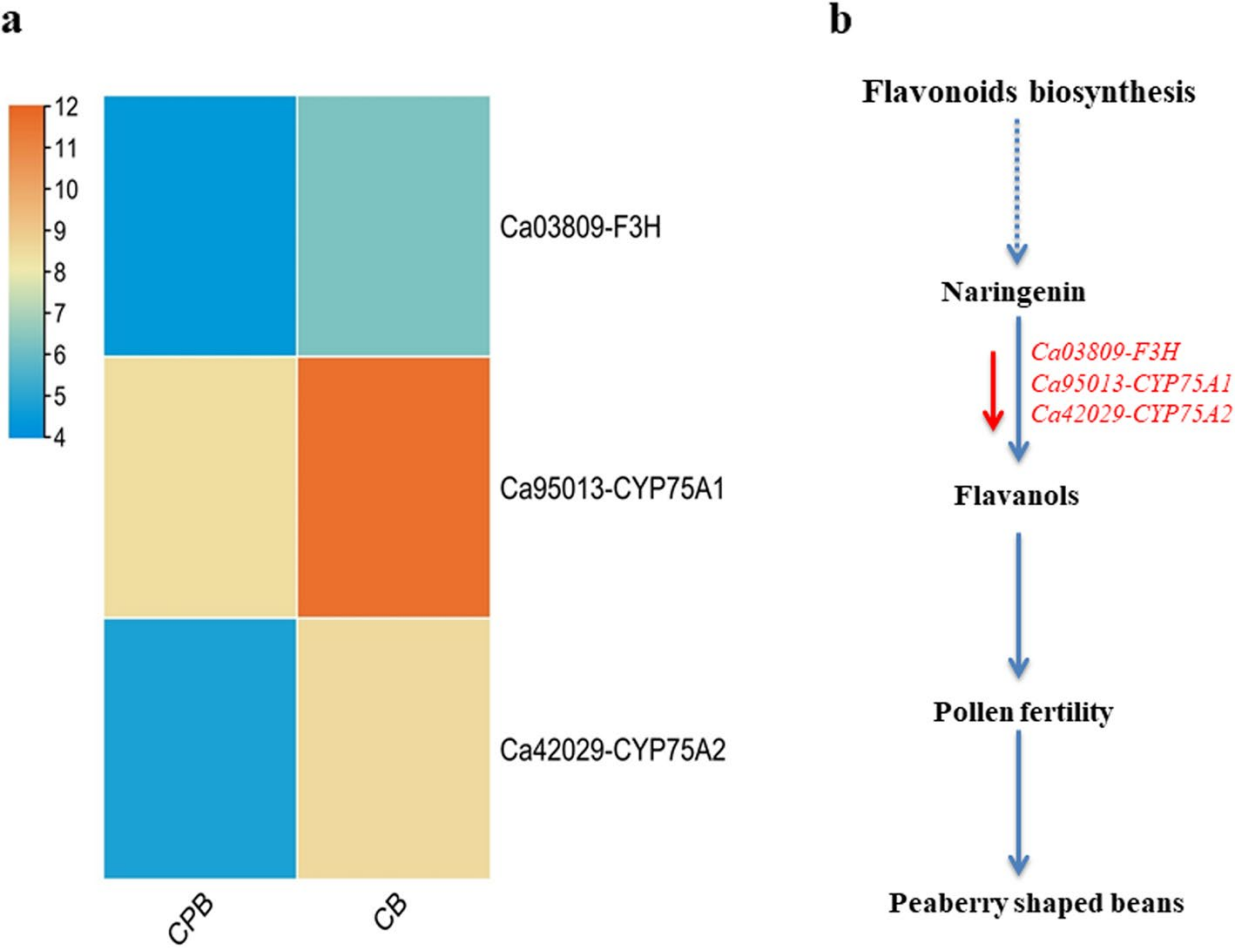
The expression profiles of flavonoid biosynthesis genes and how these influence the bean shape of peaberry **a** Expression profiles among CPB and CB **b** Simplest predicted mechanism of peaberry-shaped beans. The lower expression of flavonoid biosynthesis genes in CBP than CB may disturb pollen fertility that results in peaberry-shaped coffee beans. The red arrow represents the down-regulation of expression. F3H: flavanone-3-hydroxylase, CPY75A: cytochrome P450 family 75 subfamily A

Flavonoids are essential secondary metabolites that comprise various subclasses in plants. The flavonoids pathway encoding genes have a crucial role in pollen growth and pollen tube formation [40]. The altered expression of genes involved in flavonoid biosynthesis is probably involved in peaberry-shaped coffee beans in Arabica (Fig. 6). Because normal fertilization leads to the independent development of two embryos into regular coffee beans whereas the maturation of only a single embryo generates peaberry coffee bean [30]. The characterization of lipid metabolism along flavonoid biosynthesis-associated genes could help to reveal how abortion of a single embryo from two embryos leads to peaberry coffee beans instead of regular beans.

### Quantitative real-time PCR (qRT-PCR) analysis

**Fig. 7:**
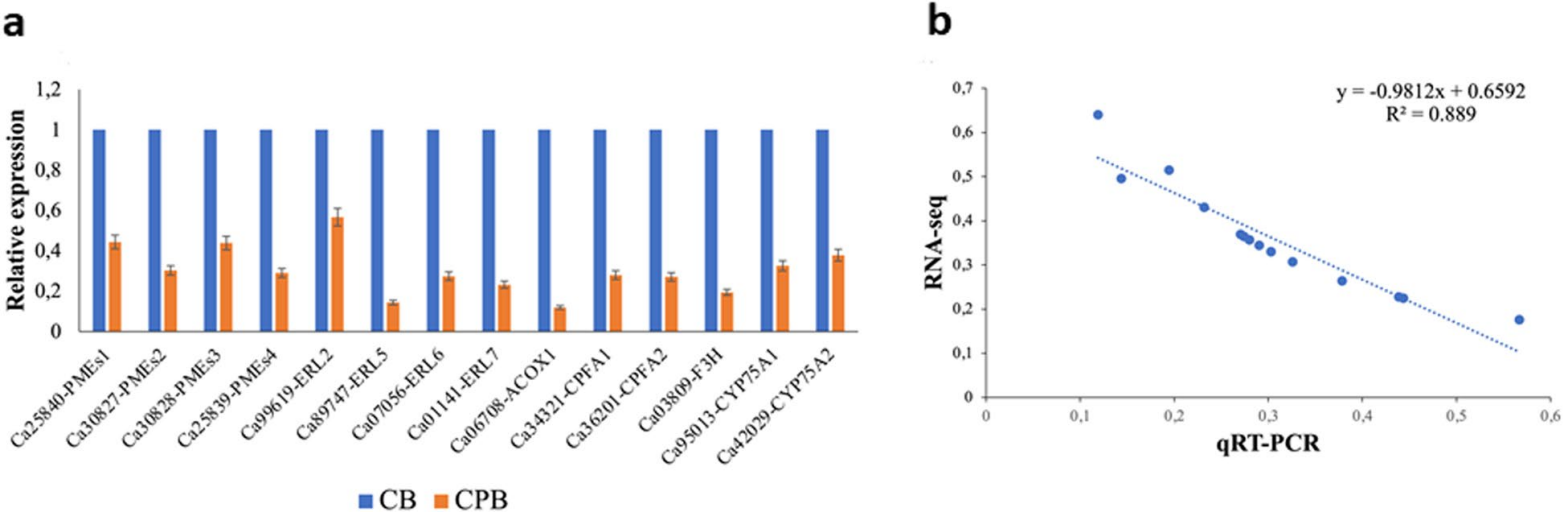
qRT-PCR analysis of 14 selected genes between CPB and CB. **a** gene expression based on the qRT-PCR approach, **b** a correlation analysis between qRT-PCR and RNA-seq expression profiles

Fourteen genes were selected to validate RNA-seq data by qRT-PCR. The selection was performed from genes associated with coffee bean quality that includes cell wall modification genes (*Ca25840-PMEs1, Ca30827-PMEs2, Ca30828-PMEs3*, and *Ca25839-PMEs4*), ERECTA protein family genes (*Ca99619-ERL2*, *Ca89747-ERL5*, *Ca07056-ERL6*, and *Ca01141-ERL7*), lipid metabolism genes (*Ca06708-ACOX1, Ca34321-CPFA1*, and *Ca36201-CPFA2*), and flavonoid biosynthesis genes (*Ca03809-F3H, Ca95013-CYP75A1*, and *Ca42029-CYP75A2*). All selected genes showed significant down-regulation in peaberry coffee beans compared to normal coffee beans in the qRT-PCR, which is consistent with RNA-seq data (Fig. 7). This result confirms the precision of the RNA-seq results in peaberry coffee beans.

## Discussion

### Influence of bean physical attributes on quality of peaberry coffee

Coffee is one of the most beverages consumed worldwide. Among all coffee species, Arabica is the most often used species due to its prime quality, taste, and flavor. It originated in Ethiopia and become a significant foreign exchange earning source for many tropical countries [41]. Plenty of research has revealed the biochemical composition of quality coffee. Usually, the quantity of peaberry bean formation is mainly low in Arabica plants but their cup quality is superior to regular beans of the same cultivars. Despite its high economic value, the molecular mechanism of peaberry coffee bean quality is not yet fully revealed. This study through comparative transcriptome analysis explored the physical and transcript difference between peaberry and regular coffee beans, identified key regulatory genes, and finally discussed the molecular mechanism of bean quality characters in peaberry coffee beans. Our phenotypic analysis found that peaberry had diverse bean physical attributes compared to regular coffee beans. The size, length, and width of a single bean were higher in regular coffee beans as compared to peaberry. The phenotypic traits can be utilized to perform grading of coffee beans before marketing. In routine practice, the peaberries and regular coffee beans must be distinguished to yield high grade coffee. The market price of peaberries is much higher than regular beans because the majority of people desire to consume rarely produced peaberry coffee beans. This result suggests that larger bean traits do not necessarily produce high quality coffee. In recent years, the international market demands superior beans to generate the best quality beverages from coffee beans [42]. The superior quality in coffee is determined by several factors that influence the final taste, aroma, and flavor of the coffee cup. These factors include the physical attributes and biochemical composition of the green coffee beans. Among bean physical attributes, the bean shape, weight, length, and width dramatically disrupt the market price as well as total time required for beans roasting [43]. The bean categorization based on physical attributes ultimately brought high market prices while mixed sized bean lots have been least chosen by the customer. Moreover, the difference in bean physical traits often leads to uneven grain roasting. It has been reported that the uncontrolled roasting process altered the visual appearance, texture, and chemical composition of coffee beans [44]. In addition, research evidence showed that bean physical attributes can be changed with the genetic makeup of cultivars, species, and genotypes interaction with the surrounding environments [20]. Thus, understanding the gene regulatory mechanism of bean quality traits is critical to harvest high grade coffee beans similar to peaberries.

### Insights on the regulatory mechanism of bean quality traits in peaberry coffee

The coffee belongs to the albuminous dicot bean crop. The bean endosperm is living tissue that contains several biochemical components. The cell wall polysaccharides, sucrose, lipids, proteins, caffeine, and chlorogenic acids are the main components of matured coffee beans [45]. The composition and concentration of these storage compounds determined the final quality index of coffee. The appropriate level of storage compound is mandatory to improve consumer physical health whereas their toxic level caused several disorders in addition to certain diseases. For example, the high caffeine consumption results in cardiovascular disorder, depression, and loss of concentration [46]. For that reason, gene networks correlated with biosynthesis and degradation of bean storage components can accelerate breeding for high quality coffee genotypes. Our comparative analysis determined significant gene expression variations among peaberry and regular coffee beans. In particular, several genes involved in regulation of storage of cell wall components, protein, and lipids were detected with dynamic expression differences. Almost, half of the total dry weight of beans is comprised of cell wall polysaccharides in coffee. The galactomannans are most abundant followed by arabinogalactans, and cellulose in green coffee beans [47]. These cell wall polysaccharides undergo complex changes during bean formation, performed specialized functions, and influence coffee flavor [48]. Besides, pectin methylesterases regulate pollen development by influencing the separation of pollen tetrads in plants [35]. Thus, altered expression of several genes involved in cell wall modification through pectinesterase as well as cellulose synthesize activity may pave the way for differential cell wall deposition that may change cell wall thickness which led to formation of peaberry coffee beans in Arabica. Furthermore, altered expression of cell wall modification related genes may regulate the quality difference of roasted peaberry coffee beans due to dissimilarity in cell wall polysaccharides deposition and degradation with the combination of other storage compounds including protein, lipids, and sugars [49]. Interestingly, the ERECTA-family receptor-like proteins influenced flower development that specifically includes anther and ovule differentiation in plants [36]. Consistent with these findings, significant lower expression of ERLs related genes in Arabica might fertilized only one embryo instead of two, which further developed to generate peaberry like coffee beans. On the other way, the modified expression profiles of peptide/histidine transportation encoding genes might cause breakdown of reducing sugars and cell wall polysaccharides into different aromatic compounds in coffee [19]. These precursor of aromatic compounds leads to the biosynthesis of different aromas which influenced the color, caramel, sweet, and burnt type aromas of coffee beans [50].

**Fig. 8:**
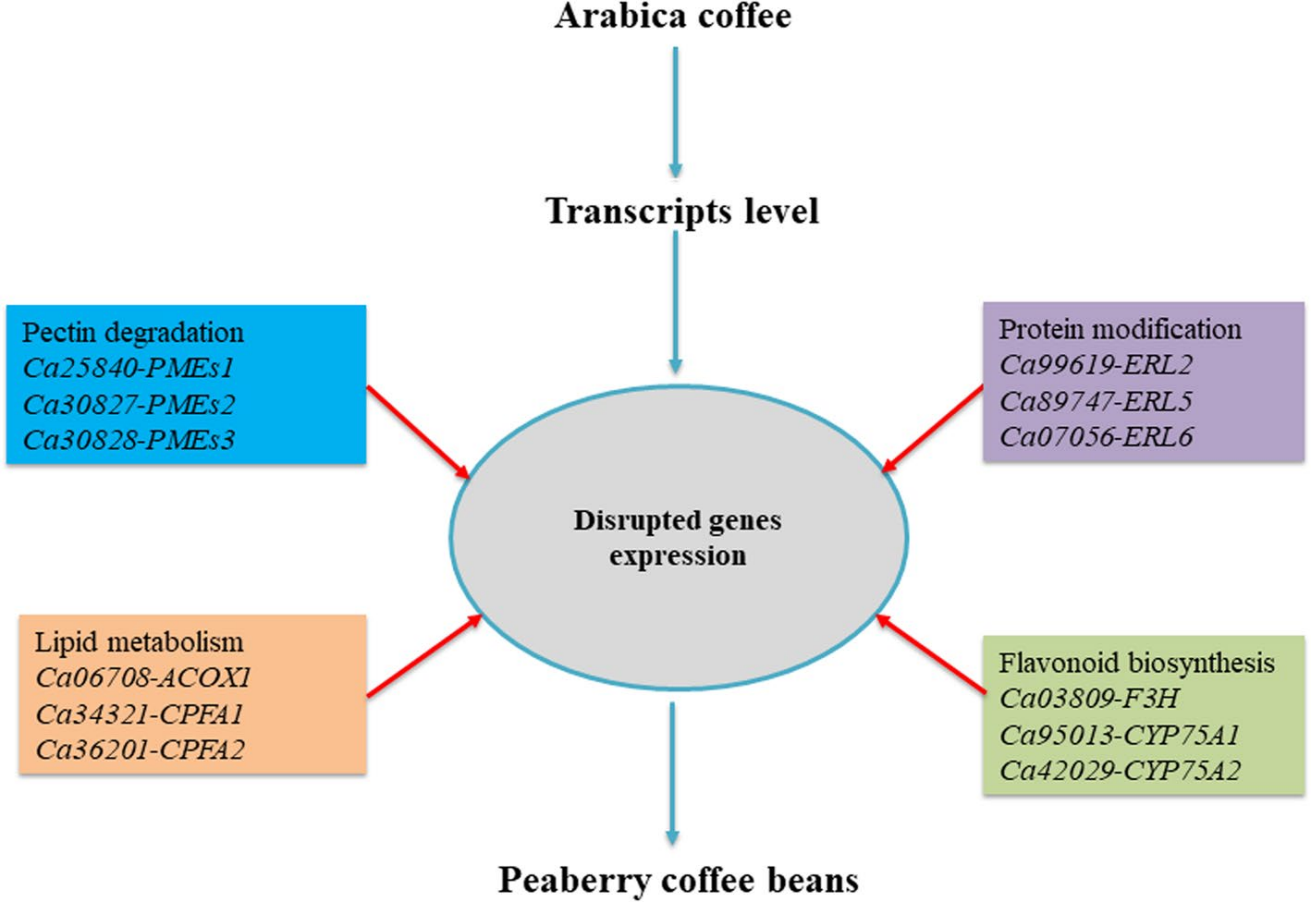
The proposed molecular mechanism for the formation of peaberry coffee beans in Arabica

The mature endosperm of coffee has 7–17% lipids in beans that consist of more fractions of triacylglycerols, fatty acids, and diterpene esters with a low level of tocopherols, phospholipids, free sterols, and wax [16]. The total lipid contents were found higher in Arabica coffee beans than in Robusta coffee beans. The previous research show mechanism of roasting has the least influence on the composition of most coffee lipids. The lipids in this way contribute to bean development, texture, flavor, and soluble vitamins in coffee [51]. Significant altered expression of lipid binding genes implies their crucial role in the storage of various types of lipids in coffee beans. Furthermore, lipid associated genes dynamic changes may control reproductive aspects of peaberry coffee beans. Since the fertility of anther and pollen declined with disruption of lipid metabolism in plants [39]. The generation of rarely formed peaberry coffee beans might be the result of an imbalance in lipid metabolism. However, further research is needed to identify the candidate genes tightly interlinked with the reproductive development of peaberry coffee beans. Previous research has shown that the coffee bean has higher percentage of saturated fatty acids than other tropical bean crops [16]. The fatty acid profiles have closely been associated with oxidative changes during the process of roasting in bean crops. The undesired oxidative changes not only adversely affect oil contents but also generate unfavorable changes in aromatic compounds [52]. The significantly lower regulation of fatty acid encoding genes in peaberry coffee may be not damaged by oxidative stress and in this way mediates additional aroma products. In plant, flavonoids are major secondary metabolites, belong to different types of flavones, flavanones, chalcones, flavonols, naringenin, and anthocyanins, and involved in several biological functions. In particular, sexual reproduction that includes pollen fertility, pollen growth, and pollen tube development is influenced with the abundance of flavonoids components in crops [40, 53]. In this regard, the significant lower expression of flavonoids biosynthesis pathway genes in peaberry indicates low abundance of flavonoids components. The lower abundance of flavonoids may disturb energy balance, reduce pollen fertility, and ultimately contribute to hardly form peaberry coffee beans. However, transgenic research can be useful to fully confirm the contribution of flavonoids components in formation of peaberry beans in Arabica. The genes involved in transmembrane transportation play a critical role in bean development. It mobilized the overall nutrient traffic, contribute to the deposition of storage components, and eventually influenced the beverage quality of coffee [54]. The different expressions of transporter genes might result in different physical and biochemical quality traits of peaberry coffee beans. In concise, integration of our research with those previously reported, we presumed that genes correlated with biosynthesis, degradation, and storage of major bean components regulate the quality attributes of peaberry coffee beans (Fig. 8). But, the potential mechanism of how these genes interact to influence the quality characters of peaberry coffee beans demands further functional genomic research with combined metabolomics and transcriptomics analyses.

## Conclusion

This study detected dissimilarity in the physical attributes of peaberry and regular coffee beans. The comparative transcriptome analysis revealed a low number of gene expression differences among both coffee beans. Specifically, the genes involved in the regulation of cell wall polysaccharides, lipids, fatty acids, proteins, and Flavonoids had dynamic expression changes. These genes most likely not only mediate different bean shape patterns but also influenced the bean composition in peaberry. Our results identified many putative candidate genes related to different bean formations in Arabica. Furthermore, provide a platform to explore the genetic mechanism of rarely formed peaberry coffee beans.

## Methods

### Plant material, phenotypic analysis, and RNA sequencing

The plant material investigated in this study was a popular *C. Arabica* variety introduced from Ethiopia (without a local name). This variety is able to provide 20% of peaberry coffee beans (CPB). The fresh fruit cherries of CPB and regular coffee beans (CB) were harvested from planting areas of Baoshan city of Yunnan province in China. The formal identification of the plant material has been conducted by Prof: Jinhuan Chen. No permission is needed to collect/study this material and a voucher specimen can be obtained at Institute of Tropical and Subtropical Cash Crops under the accession number: ITSCC4296100X.

After sample harvesting, 20 beans were randomly selected each for CBP and CB. The fruit was peeled before the determination of single grain weight (g), bean length (mm), and bean width (mm). The high quality RNA was extracted in three biological replicates for each coffee bean by using TRIZOL® reagent (Life Technologies, Carlsbad, CA, USA). The RNase-free DNase I (TaKaRa, Kyoto, Japan) was mixed to remove genomic DNA contamination. The RNA concentration and purity were later confirmed with NanoDrop ND-1000 (NanoDrop, Wilmington, DE, USA). The accurate detection of RNA integrity was accessed with Bioanalyzer 2100 (Agilent Technologies, California, USA). After preliminary quality measurements, the poly (A) RNA was fragmented into small pieces using Magnesium RNA Fragmentation Module (NEB, cat.e6150, USA). The cleaved RNA fragments were then reverse transcribed to synthesize six individual final cDNA libraries according to the protocol for the mRNA-Seq sample preparation kit (Illumina, San Diego, USA). The agarose gel electrophoresis was used for final fragment size selection and then PCR amplification was performed with standard protocol. After final libraries were constructed with standard quality, pair-end RNA sequencing was performed on Illumina HiSeq 4000 platform with recommended protocol at Wuhan Baiyi Huineng Biotechnology Co., Ltd China.

### Transcriptome data analysis

The raw sequenced data were acquired from RNA sequencing platform. The high quality clean reads were produced from raw reads by filtering low quality reads, adaptors, and ambiguous bases with FASTQ software [55]. Clean reads were aligned with the coffee reference genome using HISAT2 [56]. Only mapped reads without mismatches were retained for transcriptome downstream analysis. The expression abundance of each gene in FPKM (fragments per kilobase of exon per million mapped fragments) form was measured with StringTie [57]. The FPKM of 0.1 was considered the threshold criteria for gene expression. The total number of differentially expressed genes (DEGs) was detected with DESeq2 [58]. The criteria log2 (fold change) ≥ 1 or ≤ -1 and *p-value* ≤ 0.05 was applied to identify DEGs between CPB and CB. Principal component analysis was performed with ggfortify package in R by using FPKM values. Pearson correlation coefficient was used to measure the correlation between samples. All DEGs were subjected to functional enrichment analysis with ClusterProfiler [59] with a *p-value ≤ 0.05* is used as the threshold for screening significant enrichment results.

### qRT-PCR analysis

The TransScript One-Step gDNA Removal kit long with cDNA SynthesisSuperMix (TransGen, China) for used to synthesize cDNA for qRT-PCR of selected genes. The gene specific primers were designed with the Oligo 7 (Table S2). The reaction mixture was prepared with QIAGEN SYBR Green PCR Kit in three biological and technical repeats for each target gene. The running protocol for qRT-PCR was followed as detailed in the previous study [60]. *Actin7* was the reference gene and the relative expression of target genes was determined with the 2^−ΔΔCt^ data analysis method.

## Supplementary Information

**Additional file 1: Figure S1.** The mapped region’s statistics for among peaberry and regular coffee beans (a) Mapped regions for peaberry coffee beans (b) Mapped regions for regular coffee beans. **Figure S2.** Principal component analysis and correlation among peaberry and regular coffee beans (a) Principal component analysis (b) Correlation analysis among different coffee beans. **Figure S3.** Functional enrichment terms of DEGs detected among peaberry and regular coffee beans. 

**Additional file 2: Table S1.** Statistics of  total expressed genes with their expression ratios in peaberry and regular coffee beans.

**Additional file 3: Table S2.** Primer sequences of the selected genes for qRT-PCR.

## Data Availability

The raw RNA-seq data has been submitted to NCBI SRA under the project number PRJNA743796 (https://www.ncbi.nlm.nih.gov/bioproject/?term=PRJNA743796). The analyzed data is presented in this article.
