# Supplementary material for: Comparative transcriptome analysis in peaberry and regular bean coffee to identify bean quality associated genes
Source: BMC Genom Data. 2023 Feb 27;24:12. doi: 10.1186/s12863-022-01098-y (PMC9969625; doi:10.1186/s12863-022-01098-y)

**Figure S1**: The mapped region’s statistics for among peaberry and regular coffee beans **(a)** Mapped regions for peaberry coffee beans **(b)** Mapped regions for regular coffee beans

**Figure S2**: Principal component analysis and correlation among peaberry and regular coffee beans **(a)** Principal component analysis **(b)** Correlation analysis among different coffee beans.


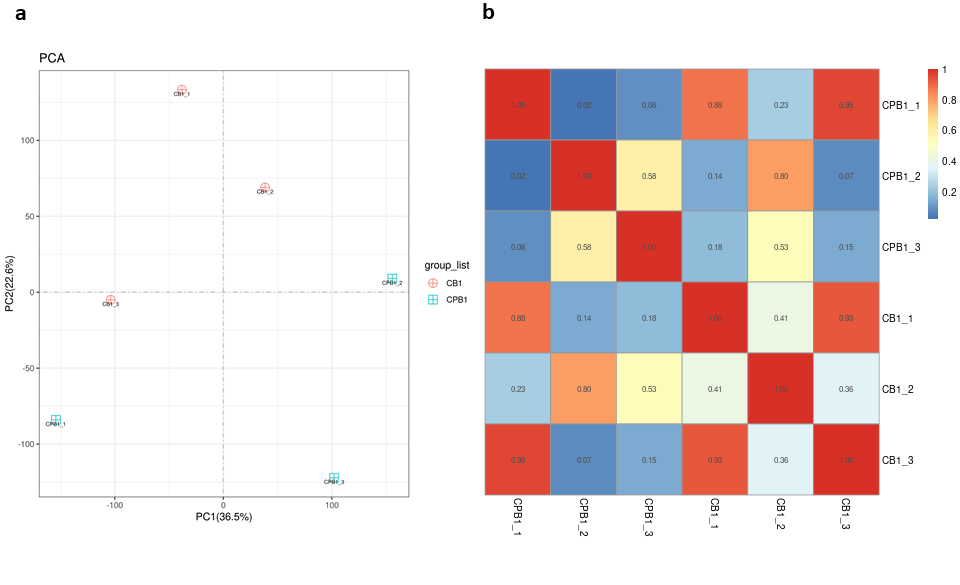


**Figure S3**: Functional enrichment terms of DEGs detected among peaberry and regular coffee beans


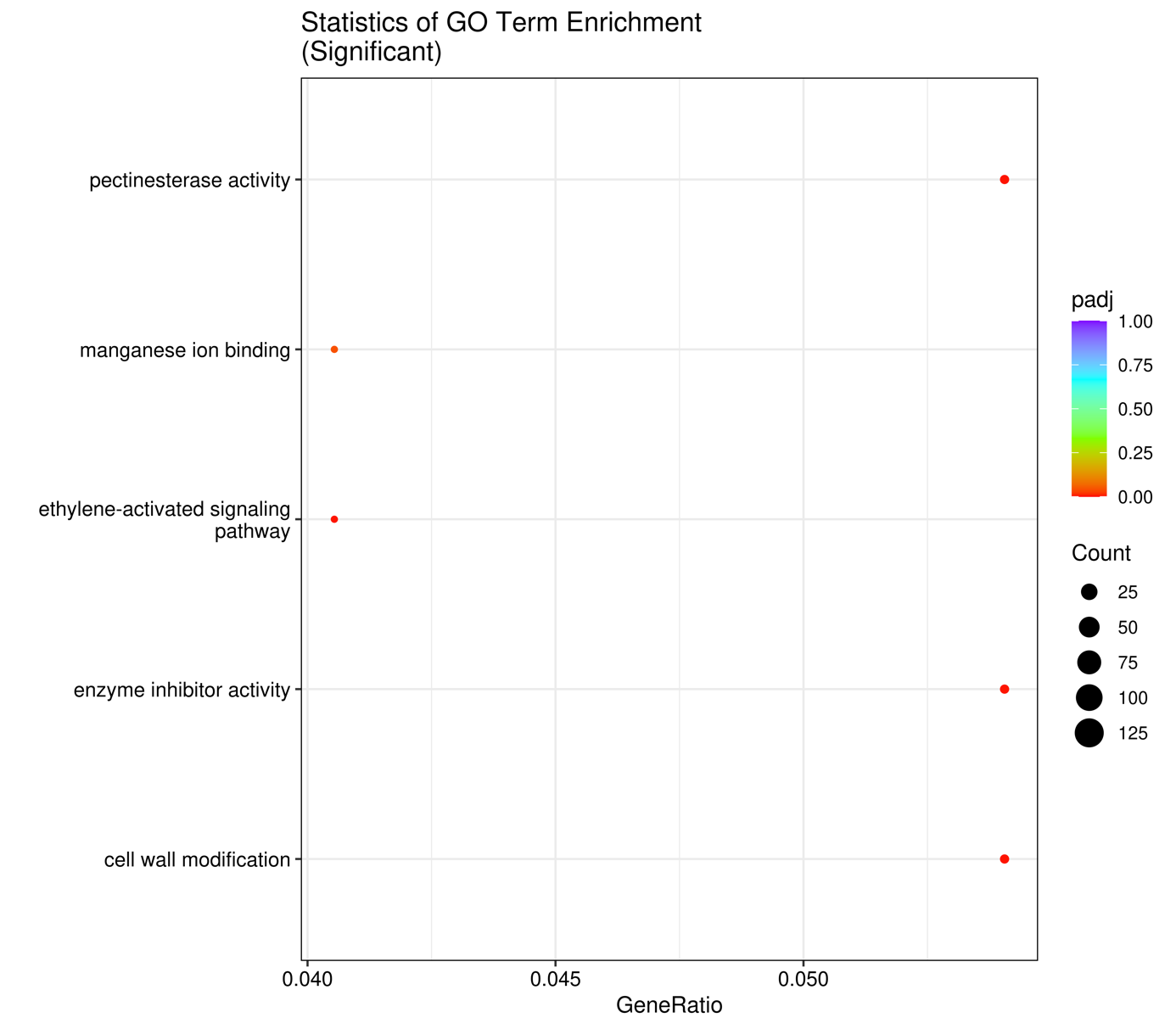

Supplement: Supplementary file 1 — Additional file 1: Figure S1. The mapped region’s statistics for among peaberry and regular coffee beans (a) Mapped regions for peaberry coffee beans (b) Mapped regions for regular coffee beans. Figure S2. Principal component analysis and correlation among peaberry and regular coffee beans (a) Principal component analysis (b) Correlation analysis among different coffee beans. Figure S3. Functional enrichment terms of DEGs detected among peaberry and regular coffee beans. [file 12863_2022_1098_MOESM1_ESM.docx]
