# Supplementary material for: Comparative transcriptome analysis in peaberry and regular bean coffee to identify bean quality associated genes
Source: BMC Genom Data. 2023 Feb 27;24:12. doi: 10.1186/s12863-022-01098-y (PMC9969625; doi:10.1186/s12863-022-01098-y)
Supplement: Supplementary file 2 — Additional file 2: Table S1. Statistics of total expressed genes with their expression ratios in peaberry and regular coffee beans. [file 12863_2022_1098_MOESM2_ESM.docx]

**Table S1:** Statistics of total expressed genes with their expression ratios in peaberry and regular coffee beans

| Sample | Total expressed genes | 0.1-3.75 FPKM | 3.75-15 FPKM | >15 FPKM |
| --- | --- | --- | --- | --- |
| CB | 38543 | 28.69% | 22.14% | 14.68% |
| CPB | 37765 | 27.63% | 20.71% | 14.33% |
